# Preparation of ELISA and Lateral Flow Kits for rapid Diagnosis of *Mycoplasma gallisepticum* in Poultry

**DOI:** 10.1038/s41598-020-65848-7

**Published:** 2020-06-03

**Authors:** Heidy Abo Elyazeed, Nayera M. Al-Atfeehy, Rasha Abotaleb, Rafik Sayed, Sherif Marouf

**Affiliations:** 1 Department of Microbiology, Faculty of Veterinary Medicine, Cairo University, Giza, Egypt; 2 Department of bacteriology, Reference Laboratory for Veterinary Quality Control on Poultry Production, Animal Health Research Institute, Agriculture Research Center (ARC), P.O. Box 264-Dokki, Nadi El-Seid st., Giza, 12618 Egypt; 3 Central Laboratory for evaluation of veterinary biologics (CLEVB), Agriculture Research Center (ARC), Giza, Egypt

**Keywords:** Immunology, Bacteriology

## Abstract

Avian mycoplasmas were mainly the cause of poultry industry economic losses; reduced meat and egg production and increases the antibiotic treatment cost. *Mycoplasma gallisepticum* (MG) infection is designated as infectious sinusitis of turkeys and chronic respiratory disease of chickens (gasping, depression, semi closed eyes, infraorbital sinuses edema and decrease in egg production). This study aimed to prepare, evaluate and Compare in-house ELISA kits and lateral flow assay (LFA) from a local strain of MG with commercial ELISA kits and PCR consequently. A total of 54 samples (27 tracheal swabs, 10 trachea and 17 lung) and 50 serum samples collected from birds suffering from chronic respiratory disease were tested by prepared in-house ELISA, commercial ELISA kits, PCR and LFA; a high correlation coefficient between in-house ELISA using whole antigen or sonicated antigen and commercial kit was recorded. Lateral Flow assay (LFA) performance indicate a low sensitivity (77.5%) but maintain a high specificity (92%) compared to PCR. The in-house ELISA kits and LFA prepared could be used as a fast diagnostic technique for detection of MG in Egypt. According to the available knowledge the prepared LFA for diagnosis of MG infection in chickens was developed for the first time in Egypt.

## Introduction

Avian mycoplasmas are a major economic burden on the poultry industry; as decreased both feed conversion and quality of carcasses at slaughter. It increase the mortality and loss of weight leading to severe economic losses. Egg transmission air and borne droplets inhalation are the main mode of disease transmission or by, resulting in rapid disease transmission throughout the flock. Since modernization, MG and MS are the most incriminated poultry pathogens and can affect breeders and broilers (gasping, depression, semi closed eyes, edema in infraorbital sinuses and decrease in egg production). MG infection is commonly denominated as chickens chronic respiratory disease (CRD) and turkeys infectious sinusitis. MS tagged as tenosynovitis and subclinical upper respiratory infection or bursitis in chickens and turkeys^1^. *Mycoplasma* has also been isolated from geese, ducks, pigeons, Amazon parrots, quails and greater flamingos^2,3^. The success of control programs either by medication or vaccination depends on accuracy and the time limit for diagnosis of infected flocks to prevent dissemination of infection^4^. Culture, serology and molecular methods are a diagnostic tools for diagnosis of avian mycoplasmas. Culturing of mycoplasmas are difficult, requiring time and complex different types of media and technical expertise; so, PCR and serology methods are much accepted and faster than culturing^5^.

Different serological methods used for identification of avian mycoplasmas as ELISA and lateral flow assay. ELISA is a rapid serological test used for detecting and quantifying antibodies or antigens. ELISA plate coating antigen prepared from local isolate is cheap and produced equally reliable results to their commercial companion^4^. Lateral flow assay is among the most fast growing qualitative and quantitative strategies for analysis. Lateral flow immunoassays are used widely in many aspects due to its advantages; the broad range of applications, the nature of the technology and low cost. it used in as clinical veterinary laboratories, hospitals, environmental assessment, and food safety production^6^.

The following work aims to prepare and evaluate in-house ELISA kits and lateral flow assay (LFA) from a local strain of MG in comparison with commercial ELISA kits and PCR consequently.

## Results

### Results of in-house ELISA and commercial *Mycoplasma gallisepticum* antibody test kit (ProFlock, Synbiotics Corporation, USA) comparison

**Figure 1:**
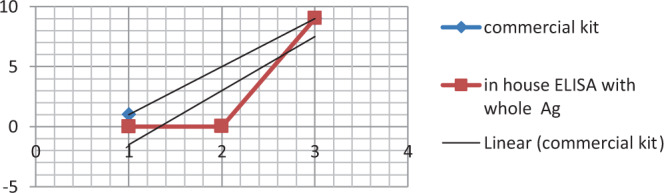
Correlation coefficient depending on the correlation of OD values between in-house ELISA (whole antigen) and commercial kits.

**Figure 2:**
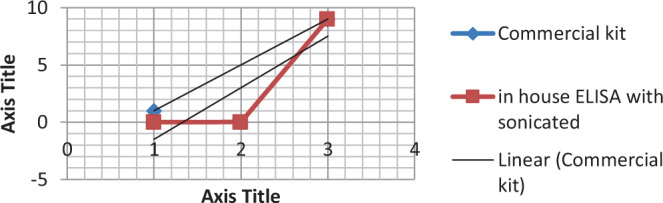
Correlation coefficient depending on the correlation of OD values between in-house ELISA (sonicated antigen) and commercial kits.

Fifty Samples were examined by in-house ELISA either coated by whole antigen or sonicated antigen of local *Mycoplasma gallisepticum* EGY1-2017 strain and commercial kits. A high correlation coefficient (Pearson’s correlation coefficient (*r*) = 0.80, *p* = 0.009) depending on OD correlation values was detected between the prepared in-house ELISA whole antigen and *Mycoplasma gallisepticum* antibody test kit (ProFlock, Synbiotics Corporation, USA) as explained in Fig. (1) and Table (1). A high correlation coefficient (*r* = 0.79, *p* = 0.011) was also observed as shown in Fig. (2) and Table (2) based on OD values obtained by in-house ELISA sonicated antigen and *Mycoplasma gallisepticum* antibody test kit (ProFlock, Synbiotics Corporation, USA). The obtained results depending on statistical analysis (Pearson correlation coefficient) indicated that plates coated with whole antigen or sonicated antigen gave similar results and good affinity for binding or adsorbing to the surface of polystyrene wells of the microtiter plate, that mean both are efficient tools as commercial one.

**Table 1:** Comparison between commercial ELISA and in-house whole antigen ELISA kits using Pearson correlation coefficient.

		Commercial ELISA	In-house ELISA whole antigen
Commercial ELISA	Pearson Correlation	1	0.803^**^
	Sig. (2-tailed)		0.009
	N	50	50
In-house ELISA whole antigen	Pearson Correlation	0.803	1
	Sig. (2-tailed)	0.009	
	N	50	50

^**^Correlation is significant at the 0.01 level (2-tailed).

**Table 2:** Comparison between commercial ELISA and in-house sonicated antigen ELISA kits using Pearson correlation coefficient.

		Commercial ELISA	In-house ELISA sonicated antigen
Commercial ELISA	Pearson Correlation	1	0.790^*^
	Sig. (2-tailed)		0.011
	N	50	50
In-house ELISA sonicated antigen	Pearson Correlation	0.790^*^	1
	Sig. (2-tailed)	0.011	
	N	50	50

**Correlation is significant at the 0.05 level (2-tailed).

### Detection of *Mycoplasma gallisepticum* using PCR

**Figure 3:**
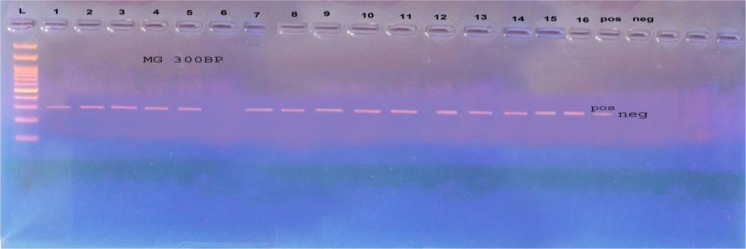
Amplified PCR product of molecular size of 300 bp using primer of *mgc*2 gene of *Mycoplasma gallisepticum*. L: Gel Pilot 100 bp ladder. 1–16: positive *mgc*2 gene of *Mycoplasma gallisepticum* except 6 was negative. pos: Control positve *Mycoplasma gallisepticum*. neg: Control negative.

A total of 31 samples out of 54 examined samples were positive for detection of *mgc2* gene of MG using conventional PCR with an incidence of 57% (Fig. 3).

### Detection of *Mycoplasma gallisepticum* using prepared Lateral Flow kits

**Figure 4:**
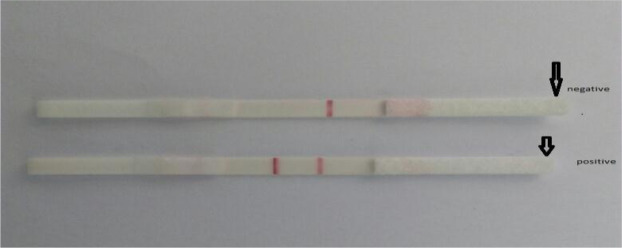
Result of detection of *Mycoplasma gallisepticum* using the developed lateral flow kits. The upper strip showing negative result. The lower strip showing positive result.

A total of 23 samples out of 54 examined samples were positive using prepared Lateral Flow kits (LF) with an incidence of 42.6%. The positive sample gave two red lines (test and control line), while the negative sample gave single control red line (Fig. 4).

### Comparison between prepared lateral flow kits and PCR results using values of K test f weighted kappa statistic (k test)

**Figure 5:**
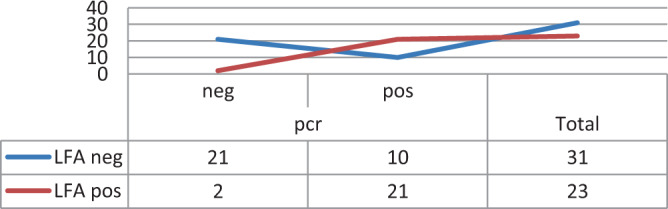
Comparison between LF kits and PCR results. LFA: Lateral flow assay. PCR: polymerase chain reaction.

The study used the calculation of the weighted kappa statistic (K test) to compare between prepared Lateral Flow kits and PCR results, K value was 0.565 that indicate moderate agreement correlation between LFT kits and PCR results as shown in Table 3 and Fig. (5).

**Table 3:** Weighted kappa statistic (K test) for correlation between local prepared Lateral Flow kits and PCR results.

		Value	Asymp. Std. Error^a^	Approx. T^b^	Approx. Sig.
Measure of Agreement	Kappa	0.565	0.106	4.339	0.000
N of Valid Cases		54			

^a^Not assuming the null hypothesis.

^b^Using the asymptotic standard error assuming the null hypothesis.

Assays performance indicate a low sensitivity (77.5%) but maintain a high specificity (92%) compared to results of PCR.

## Discussion

*Mycoplasma gallisepticum* (MG) is considered the most important pathogen, which causes great economic losses within the poultry industry. Historically, detection of mycoplasma is very difficult and demanding task for many researchers and thus infection can go unnoticed^7^. Traditional culturing is time consuming as the organism is slow growing, and some are fastidious and may not be detected^8^. Serology is much faster but non-specific reactions, cross-reactions and cost are all disadvantages. PCR seems to be an alternative rapid method, but it is more expensive. For these reasons, a rapid, simple performance, time saving and inexpensive methods of detection of the organisms are required^9^.

The cost of available ELISA kits bought from aboard usually too high and not affordable by the most diagnostic veterinary laboratories in Egypt with low resource, so the following research was designed to prepare a rapid, accurate, time saving and cheap kits for diagnosis of avian mycoplasmosis.

The results indicate that the prepared ELISA kits produce equally reliable results when compared with commercial kits. The obtained results agree with the results of Fatema *et al*.^10^ who revealed excellent correlation of prepared ELISA with two commercial kits based on OD values; Pearson 0.957, P = 0.001 for monoclonal antibody coated kit and Pearson 0.929, P = 0.000 for polyclonal antibody coated kit. Also Wanasawaeng *et al*.^11^ indicated that the ELISA prepared with field strain revealed high sensitivity and specificity in compared with the commercial ELISA test (67%, 95% respectively).

The obtained results of both whole antigen or sonicated antigen gave similar results and good binding affinity for the surface of the microtiter plates polystyrene wells, which go parallel to Optiz and Cyr^12^ results that recorded strong adsorbing affinity to the surface of the microtiter plates polystyrene wells at prepared the myco-ELISA antigen from disrupted *M.gallisepticum* or whole bacterial cell.

Lateral Flow assays (LFAs) have attracted attention to many researchers due to their high specificity, accuracy, low cost, high sensitivity and easy application by non-specialized personnel.

For the identification of *Mycoplasma gallisepticum* using developed Lateral Flow kits, a total of 23 samples out of 54 examined samples were positive for MG with an incidence of 42.6%.The samples were previously tested using conventional PCR.

Comparison between LFA kit and PCR results, K value was 0.565 that indicate moderate agreement correlation between LFA kit and PCR results as a monitoring farms methods. The developed Lateral Flow kits gave results in five minutes. Assays performance indicate a low sensitivity (77.5%) but maintain a high specificity (92%) compared to PCR, while Hasan *et al*. (2016) recorded that The sensitivity and specificity of LFA kit compared to PCR was found to be 56.6% and 91.18%, respectively. In conclusion, the prepared in-house ELISA with either whole or sonicated MG antigen is of diagnostic value in comparison with commercial one regarding to its low cost. Also LFA is fast, highly accurate and more sensitive method for evaluation and detection of MG from suspected cases. According to the available knowledge the prepared lateral flow kits for diagnosis of MG infection in chickens was developed for the first time in Egypt.

## Materials and Methods

### Strain used

A local *Mycoplasma gallisepticum* strain EGY1-2017 with accession number (MG742314) was used in this work.

*Mycoplasma gallisepticum* strain derived from Animal Health Research Institute, Doki, Giza, Egypt, used as positive control for both PCR and lateral flow assay.

### Ethical approval

All animal related contact procedures were carried out with relevant guidelines and regulations by Veterinary Cairo University institutional animal care and use committee (Vet.CU-IACUC), according to local Egyptian laws. The study were approved ethically with document number Veterinary Cairo University-10102019092.

### Samples used

A total of 54 samples (27 tracheal swabs, 10 trachea and 17 lung) collected from birds suffering from chronic respiratory disease (gasping, respiratory distress and facial edema) were used for PCR test and LFA.

Fifty serum samples collected from the previously described birds were used in in-house ELISA and commercial ELISA test.

### Preparation of in-house ELISA

Whole cell *Mycoplasma gallisepticum* antigen was prepared according to Avakian *et al*.^13,14^. The prepared *Mycoplasma gallisepticum* antigen (either whole antigen or sonicated) was diluted by carbonate – bicarbonate buffer (pH 10) to 1:100 (1.5 mg/ml protein) and coated to polystyrene plates (one by whole antigen and the other by sonicated antigen). The antigen – coated plates were incubated overnight at 4 °C. The antigen coated plates were washed three times with washing buffer for 5 minutes each time. All liquid must be removed after the final wash. The tested serum samples, reference positive and negative serum were diluted with blocking buffer (pH 7.1) at 1:100 ranges. One hundred µl of the diluted serum samples were added in the appropriate well and incubated for 2 hours at 37 °C in an incubator shaker. The plates were then washed three times with washing buffer for 5 minutes each time. One hundred µl of Horse Radish peroxidase conjugate lgG diluted to 1:2500 were added to each well and incubated at room temperature overnight, the plates were washed again three times. One hundred µl of OPD were added then incubated for 15 minutes at room temperature. The plates were then read at wavelength 492 nm. Cut- off point (OD = 0.5 or >0.5 was positive and <0.5 was negative), which was calculated according to Baseman *et al*. (2004) as follow: Cut- off point = mean of negative control + 3 (St.Dv).

### Commercial *Mycoplasma gallisepticum* antibody test kit (ProFlock, Synbiotics Corporation, USA)

used for comparison with in-house ELISA.

### Polymerase chain reaction (PCR) for *Mycoplasma gallisepticum* using *mgc2* primer

A total of 54 samples were examined by PCR as a gold standard for *Mycoplasma* spp. using *mgc2* primer^15^.

### Preparation of lateral flow technique for *Mycoplasma gallisepticum*

The same 54 samples were mixed with application buffer (30 mM Tris, 336 nM NaCl, 9 mM EDTA, 1% Tween 80. pH to 9.3) before examined by the local prepared lateral flow technique and compared with the result of PCR. The local lateral flow kits were prepared as the following:

#### Preparation of polyclonal antibodies (PAb) against whole MG antigens in rabbit

Antigens with or without Freund complete adjuvant (Difco) were described by Avakian *et al*.^14^. The immunization schedule was based on that suggested by Morton and Roberts^16^.

#### Purification of PAb from Rabbit using Caprylic acid

About 25 ml of serum was centrifuged for half hour at 10000 × g, then discarded the pellet.Add serum volume of 0.06 M sodium acetate buffer (pH 4.6) and put on a magnetic stirrer, then drop wise of2.02 ml of caprylic acid was dropped slowly while stirring at 22–27 °C for 30 min. Then the mixture was centrifuged at 10000 × g for twenty min, the supernatant was retained. And was dialyzed at 4 °C overnight with two or three buffer changes against PBS buffer, Finally the spectrophotometer measured the purified PAb^17^.

#### Preparation of colloidal gold (CG) nanoparticles

The authors prepared the colloidal gold nanoparticles at 40 nm diameter size. About 50 ml of ultrapure water, 0.1% HauCl4 was added. The mixture was boiled with vigorously stirring then 1 ml of 1% (w/v) sodium citrated was added quickly. the color solution was changed to red (after 2 min) and keep at the boiling temperature for another 10 min. the 0.02% (w/v) of sodium azide was added After cooling. The papered nanoparticles were scanned at range 400–600 nm using spectrophotometer to determine its diameter^18^.

#### Conjugation of the purified rabbit PAb against MG antigen with colloidal gold (CG)

0.02 M K2CO3 was used to adjust the pH of colloidal gold at 8.5.Half ml of purified rabbit antibodies (1 mg/ml) was added to fifty ml of colloidal gold nanoparticles. The mixture was stirred gently for ten min. Blocking using with PEG 20000 1% m/v final concentration then the mixture was stirred for another fifteen min. after that was centrifuged at 10.000 × g for thirty min. The conjugated pleat was diluted in 1 ml conjugation dilution buffer (20 mM Tris contain 0.02%(w/v) sodium azide 3% (w/v) sucrose and 1% (w/v) BSA) and kept at refrigerator^19^.

#### Preparation of lateral flow immunochromatographic test (LFIT)

Sample pad: of pretreated glass fiber with sample pad treated solution pH 8.5 (ultra pure water included, 2% (w/v) titronX100, 3.81% (w/v) Borax, 1% (w/v) PVP, 0.5%(w/v)sodium cholate, 0.15% (w/v) SDS, 0.1% (w/v)casein sodium salt, 0.02% (w/v) sodium azide) then dried at 37 °C^20,21^.

Conjugation pad: also made of pretreated glass fiber with conjugation treated solution pH 7.4 (2.5% (w/v) sucrose, 20 mM PBS included 0.3%(w/v)PVP, 2% (w/v) BSA, 1% (w/v) titron x100 and 0.02% (w/v) sodium azide), Then immersed with conjugation dilution buffer finally put in drier at 37 °C.

(NC): the dispenser (Isoflow, USA) was used to dispense test and control line on the nitrocellulose membrane (2.5 cm × 30 cm). Firstly dispense the rabbit antibodies (1 mg/1 ml) around the bottom a the test line (1 μl/1 cm line) then the goat anti-rabbit antibodies (0.5 mg/ml) were dispensed at the upper position as the control line (1 μl/1 cm line)the distance between control and test lines was 0.5 cmthen dried at 37 °C.The treated sample pad, immersed conjugation pad, loaded NC membrane and absorption pad were stick down in the PVC card. Cut the collected PVC card into 0.39 cm width test-strips by using cutter automating machine.

Two red line (test and control line) is considered positive sample while single control red line is considered negative sample.

### Statistical analysis (ROC analysis and k test)

The weighted kappa statistic (K test) and values of K test f weighted kappa statistic (k test) calculation were used in study, the Values of k test from 0.41 to 0.60 indicate moderate agreement; values from 0.61 to 0.80, substantial agreement, and values from 0.81 to 0.99, almost perfect agreements^22^.

Comparison of OD values of commercial and in-house ELISA kits was carried out by Pearson correlation coefficient. SPSS version 16 was used for statistical calculations.
